# Genetic control of bacterial biofilms

**DOI:** 10.1007/s13353-015-0309-2

**Published:** 2015-08-21

**Authors:** Krystyna I. Wolska, Anna M. Grudniak, Zofia Rudnicka, Katarzyna Markowska

**Affiliations:** Department of Bacterial Genetics, Institute of Microbiology, Faculty of Biology, University of Warsaw, 1 Miecznikowa Street, 02-096 Warsaw, Poland

**Keywords:** Biofilm, Cyclic diguanosine-5’-monophosphate, Quorum sensing, Small RNAs

## Abstract

Nearly all bacterial species, including pathogens, have the ability to form biofilms. Biofilms are defined as structured ecosystems in which microbes are attached to surfaces and embedded in a matrix composed of polysaccharides, eDNA, and proteins, and their development is a multistep process. Bacterial biofilms constitute a large medical problem due to their extremely high resistance to various types of therapeutics, including conventional antibiotics. Several environmental and genetic signals control every step of biofilm development and dispersal. From among the latter, quorum sensing, cyclic diguanosine-5’-monophosphate, and small RNAs are considered as the main regulators. The present review describes the control role of these three regulators in the life cycles of biofilms built by *Pseudomonas aeruginosa*, *Staphylococcus aureus*, *Salmonella enterica* serovar Typhimurium, and *Vibrio cholerae*. The interconnections between their activities are shown. Compounds and strategies which target the activity of these regulators, mainly quorum sensing inhibitors, and their potential role in therapy are also assessed.

## Introduction

**Fig. 1:**
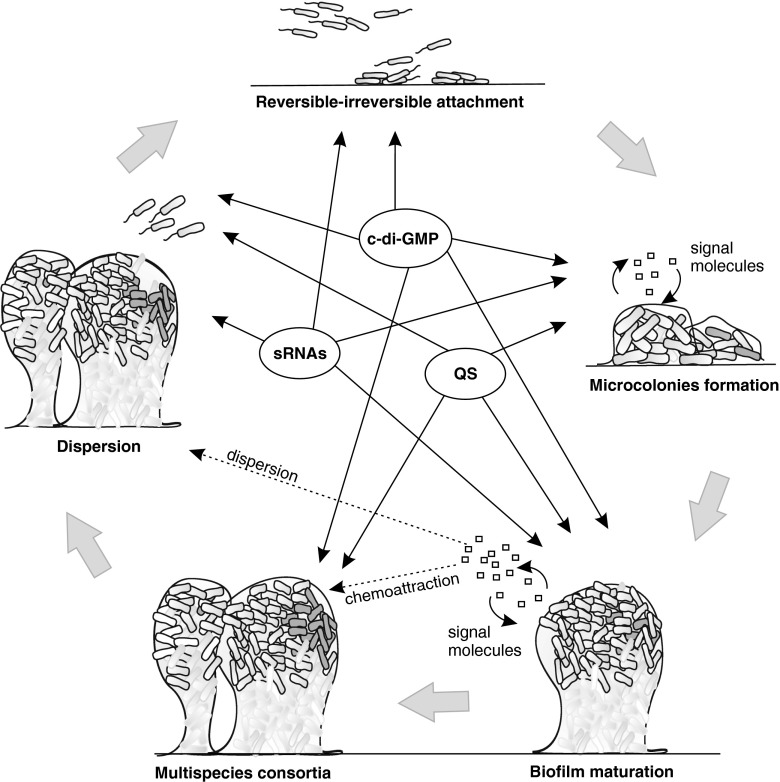
Subsequent stages of bacterial biofilm formation/dispersal and their genetic regulation. (i) reversible, followed by irreversible, attachment to the surface, (ii) formation of microcolonies, (iii and iv) biofilm maturation leading to the formation of bacterial consortia, and (v) biofilm dispersal. The regulatory involvement of quorum sensing (QS), bis-(3’-5’)-cyclic diguanosine monophosphate (c-di-GMP), and small RNAs (sRNAs) is shown by the arrows

The majority of bacteria, including clinically relevant microorganisms, are able to grow in biofilms adhering to abiotic and biotic surfaces (for a review, see Donlan and Costerton 2002). A biofilm is defined as a structured microbial community whose development requires a significant change in bacterial physiology and results in increased tolerance to exogenous stress, including treatment with antibiotics and other biocides (Hall-Stoodley and Stoodley 2009). Bacterial biofilms can form a monolayer or, most frequently, multilayers in which bacteria are attached both to the surface and to neighboring bacteria by an extracellular matrix consisting of polysaccharides, proteins, and DNA (Karatan and Watnick 2009). The biofilm formation process always has several stages that include: (i) attachment to the carrier surface, (ii) reversible, followed by irreversible, binding to the surface with the participation of adhesins, (iii) development of microcolonies, and (iv) maturation of biofilm architecture (Donlan 2001). Under unfavorable conditions, the synthesis of matrix compounds decreases and the matrix is enzymatically cleaved, leading to biofilm dispersion (Gjermansen et al. 2005). A scheme of the biofilm life cycle is presented in Fig. 1.

Biofilm formation and dispersal are highly controlled processes regulated at the genetic level and by environmental signals. Current knowledge points to quorum sensing (QS), bis-(3’-5’)-cyclic diguanosine monophosphate (c-di-GMP), and small RNAs (sRNAs) as the main regulators of bacterial biofilms, at least in several Gram-negative species (Fazli et al. 2014).

**Fig. 2:**
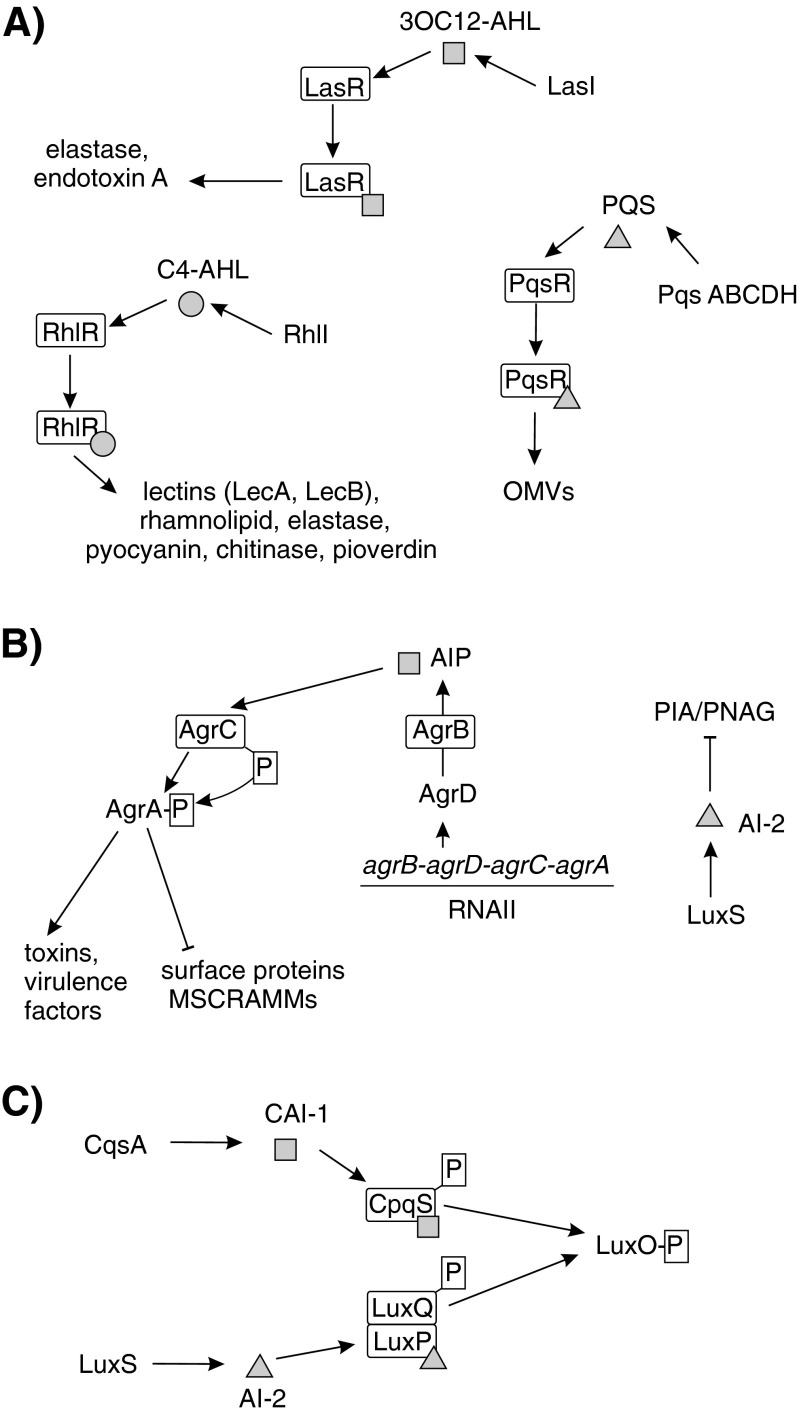
Schematic representation of QS system. **a** *Pseudomonas aeruginosa* LasI, RhlI, and PqsABCDH synthesize the QS signal molecules: N-(3-oxo-dodecanoyl)-L-homoserine lactone (3OC12-AHL), C4-AHL, and 2-heptyl-3-hydroxy-4-quinolone (PQS), respectively. The transcription factors LasR, RhlR, and PqsR detect their respective signal molecules, resulting in the regulation of target genes transcription. **b** *Staphylococcus aureus* QS peptide AIP is synthesized as a longer precursor by AgrD and is processed and secreted via AgrB. The extracellular signal is detected by the membrane-located histidine kinase AgrC and signal transduction occurs by phosphorelay to the AgrA response regulator. In the second QS system, LuxS synthesizes AI-2, which inhibits PIA/PNAG exopolysaccharide synthesis through an unknown QS cascade. **c** *Vibrio cholerae* LuxS and CqsA synthesize AI-2 and CAI-1 signal molecules, respectively. These signal molecules are detected by their corresponding receptors, the two-component histidine kinases LuxPQ and CpqS, which transfer phosphate residue to transcription activator LuxO. The regulation by LuxO–P is presented in Fig. 5

QS is considered a special “language” used for intercellular communication, which is based on small, self-generated signal molecules called autoinducers. When sufficient bacteria are present and the concentration of autoinducers reaches a threshold level, the bacteria start to sense their critical mass and answer by repressing or activating target genes (de Kievit and Iglewski 2000). QS-controlled genes can constitute around 10 % of the bacterial genome (Wagner et al. 2003). QS systems play a very important role during the development and dispersal of bacterial biofilms. Although these systems are not involved in the attachment and initial biofilm growth stages, they are required for further biofilm development and, also, are the main regulators of biofilm dispersal (Davies et al. 1998). The QS signaling pathways in *Pseudomonas aeruginosa*, *Staphylococcus aureus*, and *Vibrio cholerae* are shown in Fig. 2.

The second main biofilm regulator, the c-di-GMP signaling network, is considered the most complex secondary signaling system discovered in bacteria. However, its complexity varies significantly and this type of signaling is absent in some bacteria (Römling and Balsalobre 2012). After binding to a variety of cellular receptors, c-di-GMP controls bacterial transcription, the activity of enzymes, and even the functioning of larger cellular structures (Hengge 2009). c-di-GMP plays a crucial role in the bacterial decision between planktonic and biofilm-associated lifestyle (Jenal and Malone 2006). The factors regulated by c-di-GMP and important for three-dimensional biofilm structure development are: synthesis of exopolysaccharides, adhesive pili and adhesins, secretion of extracellular DNA (eDNA), and also control of cell death and motility. Regulatory connections between QS and c-di-GMP have been proved; it has been shown that cell density itself is one of the environmental cues sensed by the c-di-GMP network (Strivastava and Waters 2012).

Finally, small non-coding RNA molecules, sRNAs, including riboswitches, have been shown to participate in post-transcriptional gene regulation in bacteria, involving a range of metabolic processes, adaptation to stress, and microbial pathogenesis (for a review, see Michaux et al. 2014; Mandin and Guillier 2013). Therefore, sRNA regulators have become powerful tools for metabolic engineering and synthetic biology (Kang et al. 2014). However, the amount of data pointing to the role of sRNA in the biofilm life cycle is rather limited.

From among the other factors involved in biofilm formation, which are beyond the scope of this review, horizontal gene transfer (Madsen et al. 2012), alternative sigma factors (Irie et al. 2010), and toxin–antitoxin systems (Wang and Wood 2011) should be mentioned.

The control of bacterial biofilm has been studied mainly in members of the genus *Pseudomonas*, including the human opportunistic pathogen, *P. aeruginosa*, but the number of papers describing biofilm regulation in other bacterial pathogens is now growing exponentially. This knowledge may be useful in biofilm manipulation, control, and eradication. The present review describes the genetics of biofilm development in representative bacterial pathogens, both Gram-negative and Gram-positive. The strategies of biofilm eradication, mainly those exploiting QS control, are also discussed.

## *Pseudomonas aeruginosa* biofilms

### Brief characteristics

**Fig. 3:**
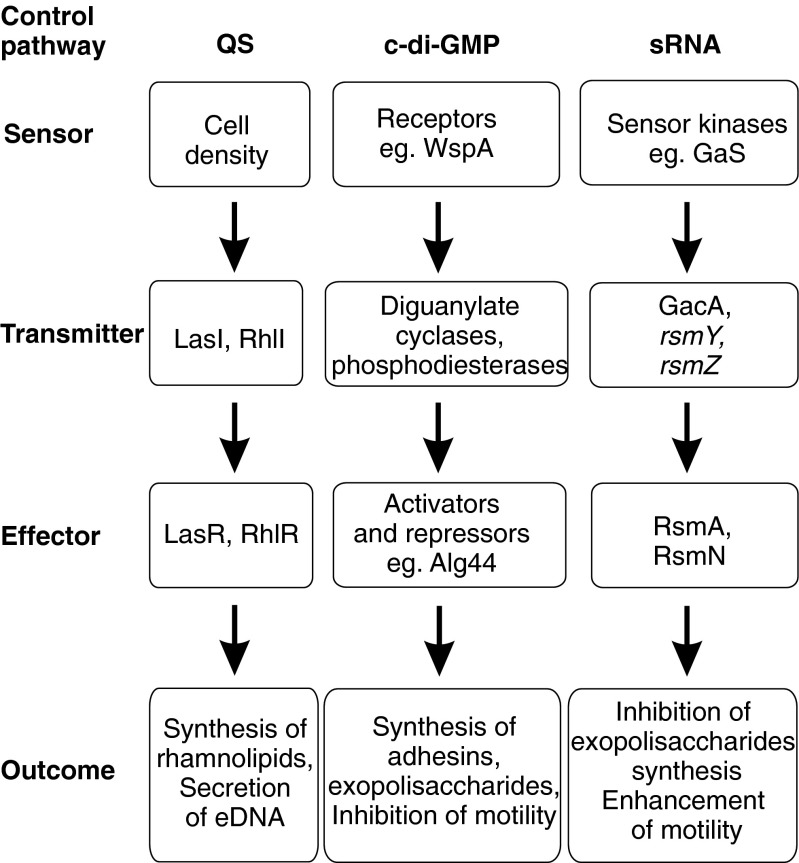
Control of biofilm formation in *P. aeruginosa*. Three control pathways are shown. In two AHL-based QS pathways, cell density plays a role of the environmental signal. The signal is then transferred through the transmitter proteins LasI and RhlI to the effector proteins LasR and RhlR, being transcriptional regulators. The QS system controls the synthesis of rhamnolipids and secretion of eDNA. In c-di-GMP signaling, the level of c-di-GMP is sensed by sensor proteins—receptors which govern the metabolism of this molecule through the activity of diguanylate cyclases (DCGs) and phosphodiesterases (PDEs). Upon binding to the effector proteins, which are activators or repressors acting at transcriptional or post-transcriptional levels, c-di-GMP controls the synthesis of adhesins and exopolysaccharides and inhibits the motility. The control by sRNA involves the activation of sensor kinases, e.g., GacS, which phosphorylates transmitter protein GacA, leading to the subsequent activation of small RNAs, inhibition of effector RsmA activity, and, finally, to the inhibition of exopolysaccharides synthesis and enhancement of motility

*P. aeruginosa* is an aerobic, non-fermenting, Gram-negative rod that has become a major opportunistic human pathogen and the leading cause of nosocomial infections in cancer, transplantation, and cystic fibrosis (CF) patients. Furthermore, due to its ability to cause chronic lung infections, this species is the primary pathogen responsible for the mortality of patients with CF (Silby et al. 2011). Many reports describe *P. aeruginosa* as being also one of the main species found in dermal and burn wounds (Ammons et al. 2009). In this species, exopolysaccharides appear to be the most important matrix components, in contrast to the other members of the genus *Pseudomonas*, *P. putida* and *P. fluorescens*, where this role is played by large surface proteins, among them the most abundant is large adhesion protein, LapA (Fazli et al. 2014). *P. aeruginosa* produces at least three secreted polysaccharides, Pel, Psl, and alginate. The last one produced by mucoid strains is considered as a *P. aeruginosa* virulence factor and, next to another compound of matrix, eDNA, seems to be of particular relevance to biofilm-mediated antibiotic resistance (Aspe et al. 2012). Three already mentioned main systems based on QS, c-di-GMP signaling, or regulatory sRNA control *P. aeruginosa* biofilm formation and dispersal. Their mode of action is described below. All systems follow the same scheme of signal transduction pathway, starting from sensors, followed by signal transmitters, and ending in effectors, which, in turn, execute the outcome, i.e., the production or modulation of factors involved in biofilm formation and detachment. The overview of these regulatory pathways is outlined in Fig. 3. The control of biofilm development in two other species of the genus *Pseudomonas*, *P. fluorescens* and *P. putida*, commonly found in soil and plant rhizosphere, and in an opportunistic pathogen, *Burkholderia cenocepacia* infecting CF patients, generally follow the outline described for *P. aeruginosa*. However, quite serious differences are observed concerning the importance of the main regulatory pathways and also the involvement of additional regulatory factors (Fazli et al. 2014).

### Quorum sensing (QS)

QS regulation of biofilm-related genes in *P. aeruginosa* in the natural environment and during persistent infections is considered as the best known example among all bacterial species (Singh et al. 2000). This bacterium has at least three QS systems: two N-acylated homoserine lactone (AHL)-based LasIR and RhlIR systems and a *Pseudomonas* quinolone signal (PQS)-based system. Both AHL systems contain a gene encoding AHL sensor/transcriptional regulator, *lasR* and *rhlR*, respectively, and a gene encoding an autoinducer, *lasI*, required for the synthesis of N-(3-oxo-dodecanoyl)-L-homoserine lactone and *rhlI* responsible for the synthesis of N-(butanoyl)-L-homoserine lactone (C4-AHL) (Pearson et al. 1994, 1995). In the PQS system, 2-heptyl-3-hydroxy-4-quinolone transported by outer membrane vesicles (OMVs) is a sensing molecule, and, in turn, PQS causes the biogenesis of OMVs (Mashburn-Warren et al. 2008; Kulkarni and Jagannadham 2014). All three systems are regulated hierarchically: LasR positively regulates RhlR and PQS and RhlR negatively regulates PQS (Pesci et al. 1997; Wade et al. 2005).

QS signaling controls the synthesis of rhamnolipids, which are important in the late stage of biofilm development, maintaining the channels in mushroom-shaped structures, resulting in the proper distribution of nutrient and oxygen and removal of waste products (Davey and O’Toole 2000). Rhamnolipids synthesis is induced in the center of biofilm mushroom caps, which is consistent with the control role of QS (Lequette and Greenberg 2005). The overproduction of these biosurfactants causes the biofilm detachment from the surface, leading to its dispersal (Boles et al. 2005). QS also plays a role in the release of a large amount of eDNA at the late stage of biofilm development, as a consequence of the autolysis of a bacterial subpopulation (Allesen-Holm et al. 2006). It is well established that autolysis is regulated by PQS, but the mechanism of this process is still not fully understood (Fazli et al. 2014). The QS system regulates the production of yet other compounds important for biofilm formation, i.e., LecA and LecB lectins (Tielker et al. 2005; Diggle et al. 2006) and siderophores pyoverdine and pyochelin (Banin et al. 2005). The last two exert their action through participation in iron metabolism; it was shown that too low or too high concentration of iron results in the inhibition of biofilm formation (Singh et al. 2002).

### c-di-GMP signaling

In general, high c-di-GMP level induces the biosynthesis of adhesins and matrix polysaccharides and inhibits various types of motility and, therefore, stimulates biofilm formation. In contrast, low c-di-GMP level downregulates the production of adhesins and exopolysaccharides and enhances bacterial motility, leading to biofilm dispersal (for a review, see Hengge 2009). Two types of enzymes control the level of c-di-GMP in bacteria. Diguanylate cyclases (DGCs), which contain typical domain, CGDEF, produce this nucleotide from two molecules of GTP, whereas c-di-GMP is broken down into 5’-phosphoguanylyl-(3’-5’) guanosine (pGpG) by specific photodiesterases (PDEs), which activity is associated with EAL or HD-GYP specific domains. The activity of both types of enzymes is controlled by environmental cues (Römling et al. 2013).

In *P. aeruginosa*, the so-called “basic c-di-GMP signaling module” consists of five components: (i) sensors of environmental signals, (ii) enzymes involved in the synthesis and degradation of c-di-GMP (see above), (iii) specific effectors which can be proteins or riboswitches, both being allosterically regulated by c-di-GMP, (iv) targets, e.g., promoter DNA, enzymes, or cellular structures such as flagellar basal body or exopolysaccharide synthetic and secretion apparatus, (v) molecular output produced by effectors after their activation by c-di-GMP (Hengge 2009).

*P. aeruginosa* contains three signal sensors (receptors), WspA, YfiB, and RocS1, which sense the level of c-di-GMP, five diguanylate cyclases, WspR, YfiN, SadC, RoeA, and SiaD, and five phosphodiesterases, BifA, DipA, RocR, MucR, and NbdA. The best characterized chemosensor, WspA, senses growth on surfaces and then becomes activated and able to phosphorylate its cognate diguanylate cyclase, WspR (Güvener and Harwood 2007). While phosphorylated, WspR forms clusters, changes the location within the cell, and its cyclase activity is increased (Huangyutitham et al. 2013). Yet another sensor, the outer membrane protein YfiB, responds to cell membrane stress and activates YfiN cyclase (Malone et al. 2012). PDEs enzymes causing biofilm dispersal are activated by such environmental cues as starvation, low concentration of oxygen, and nitric acid, but their precise mechanism of activation has not been elucidated (An et al. 2010; Li et al. 2013). The multitude of sensors, as well as c-di-GMP synthesizing and breaking enzymes, allows the c-di-GMP to be controlled by various environmental cues.

Four *P. aeruginosa* effectors, Alg44, FleQ, PelD, and FimX, regulate different targets involved in biofilm development. Alg44 activated by c-di-GMP controls the synthesis of alginate, an exopolysaccharide important in the late stages of infection with *P. aeruginosa* mucoid strains (Merighi et al. 2007). FleQ acts as both a repressor and an activator of *pel* operon encoding Pel exopolysaccharide synthesis (Baraquet and Harwood 2013), regulates the synthesis and transport of outer membrane adhesin, CdrA (Borlee et al. 2010), and represses the expression of flagellum biosynthesis genes (Baraquet and Harwood 2013). Two other effectors, PelD and FimX, regulate Pel synthesis at a post-transcriptional level and control twitching motility (Lee et al. 2007; Jain et al. 2012).

### Control by sRNA

Regulation by sRNAs, *rsmY* and *rsmZ*, is the best known example. In this pathway, the role of a sensor is played by three sensor kinases, RetS, LadS, but mainly GacS (Ventre et al. 2006). GacS phosphorylates GacA (Goodman et al. 2009), which, in turn, activates the transcription of *rsmZ* and *rsmY*. These two sRNAs are also controlled by the other proteins participating in the *P. aeruginosa* phosphorelay system (Petrova and Sauer 2010). *rsmZ* and *rsmY* reduce the activity of effector protein RsmA, being a negative post-transcriptional regulator of the biofilm matrix polysaccharide Psl (Irie et al. 2010), and also downregulate another effector, RsmN, controlling the same functions as RsmA (Marden et al. 2013). It was shown that an increased expression of *rsmY* and *rsmZ* results in enhanced initial attachment to abiotic surfaces but, on the contrary, subsequent biofilm development is hampered by the high level of these sRNAs (Chambers and Sauer 2013). Another sRNA, *phrS*, stimulates the *P. aeruginosa* PQS pathway. *phrS* expression requires global oxygen-responsible regulator ANR, which provides a regulatory link between oxygen availability and PQS (Sonnleitner et al. 2011).

The list of factors controlling *P. aeruginosa* biofilm development should be extended by the alternative sigma factor, RpoS. It was shown that, in *P. aeruginosa*, PAO1 biofilm *rpoS* expression is increased (Waite et al. 2006) and RpoS acts as a positive regulator of the expression of the *psl* gene (Irie et al. 2010). Finally, it was postulated that yet another, fatty, cis-2-decenoic acid-mediated, signaling may play a role in biofilm dispersal (Amari et al. 2013). However, the mechanism of its activity has not yet been resolved.

## *Staphylococcus aureus* biofilms

### Brief characteristics

*S. aureus* is a Gram-positive, nonmotile coccus able to form cell clusters and producing yellow pigment. This bacterium is a causative agent of acute and chronic infections. Its ecological niche in humans is the anterior nares. *S. aureus* biofilm persists on medical implants and catheters, constituting a significant healthcare problem (Kiedrowski and Horswill 2011). The list of *S. aureus* biofilm-related diseases is long and includes: osteomyelitis, indwelling medical device infections, periodontitis and peri-implantitis, chronic wound infection, chronic rhinosinusitis, endocarditis, and ocular infections (for a review, see Archer et al. 2011).

*S. aureus* biofilm life cycle follows the typical scheme already described for *P. aeruginosa*. Due to its lack of motility, the biofilm is flatter than those formed by motile genera, although mushroom forms can also be observed (Mann et al. 2009). In the biofilm, a great number of slow-growing cells and persister cells—nondividing and tolerant to antibiotics—are present (Lewis 2007). *S. aureus* biofilm is embedded within a glycocalyx or slime layer composed primarily of teichoic acid and staphylococcal and host proteins (Husain et al. 2013). The polysaccharide PIA (polysaccharide intercellular antigen), composed mainly of polymeric *N*-acetyl-glucosamine and eDNA, is also a significant biofilm constituent. The genetic control of PIA synthetic operon, *icaADBC*, involves many factors, among them the main IcaR repressor and the second TcaR repressor (Cramton et al. 1999; Jefferson et al. 2004). The expression of *icaR* gene is positively regulated by protein Spx (suppressor of clpP and clpX), a global regulator of stress response and negatively regulated by Rbf protein (Cue et al. 2009; Pamp et al. 2006). Moreover, SrrAB (staphylococcal respiratory response regulator) is responsible for PIA induction under anaerobic conditions (Ulrich et al. 2007). It was shown that biofilm formation in several *S. aureus* strains, including MRSA (methicillin-resistant *S. aureus*), does not depend on PIA production (Fitzpatrick et al. 2005), which is substituted by proteinaceous cell-to-cell adhesion with the participation of, e.g., biofilm-associated protein, Bap (Lasa and Penadés 2006). Another important component of staphylococcal biofilm, eDNA, is released as a consequence of cell lysis by holin homolog CidA and other proteins (Rice et al. 2007; Brady et al. 2006). However, massive cell lysis takes place at the late stage of biofilm development, eDNA is also released at an early stage, thus participating in cell attachment (Mann et al. 2009). In highly aggressive *S. aureus* isolates, a novel toxin family, phenol-soluble modulins (PSMs), contributes to biofilm development and dispersal and, therefore, also in the dissemination of biofilm-associated infections (Peschel and Otto 2013). It should be noted here that appreciable strain-dependent variations across staphylococcal biofilm composition are observed (Kiedrowski and Horswill 2011).

*S. aureus* biofilm development is regulated by many environmental conditions and genetic signals. From among the latter, the most important are QS and control by small molecules, including sRNA.

### Control by QS, c-di-GMP, and sRNA

The accessory gene regulator (Agr) system plays a crucial role in the functioning of the *S. aureus* QS system. In this genus, QS positively regulates toxins and acute virulence factors and negatively regulates surface proteins named microbial surface components recognizing adhesive matrix molecules (MSCRAMMs), thus inhibiting adhesion to human matrix proteins, e.g., fibrinogen (Clarke and Foster 2006; Pei et al. 1999).

QS also enhances cell detachment from mature biofilm. The *agr* locus contains *agrA*, *agrC*, *agrD*, and *agrB* genes, constituting the so-called transcript RNAII. The prepheromone AgrD is exported and modified by AgrB, which results in formation of the characteristic thiolactone-containing autoinducing peptide (AIP). AIP activates the two-component AgrC/AgrA system, which, in turn, activates the transcription of RNAII, providing an autofeedback loop (Ji et al. 1995). AIP also influences the transcription of bifunctional RNAIII regulating MSCRAMMs and encoding d-hemolysin (Fechter et al. 2014). The expression of *psm* genes coding for PSMs surfactants responsible for biofilm maturation and dispersal by the disruption of noncovalent interactions between biofilm cells and matrix components are also under the influence of the QS system (Periasamy et al. 2012; Otto 2014). The staphylococcal QS system upregulates the expression of peptidases and nucleases, which also increases biofilm detachment (Boles and Horswill 2008; Lauderdale et al. 2009). The data of several experiments support the inhibitory role of QS on biofilm development; for example, it was demonstrated that *agr* mutants form a thicker biofilm compared with wild-type strains (Vuong et al. 2003).

It was postulated that the ability to form a biofilm is closely related to the character of infection, and the determinants of acute and chronic virulence are regulated by QS in an opposite fashion. QS is important in expressing acute virulence and the formation of a differentiated biofilm with the capacity for dissemination, whereas chronic infections are concomitant with biofilm downregulation and mutation in the QS system. This postulate is supported by the observation that QS mutants are found in elevated numbers in chronic infections (Shopsin et al. 2010). However, it was recently shown that a significant fraction of *S. aureus* bacteremia cases are caused by *agr*-defective strains; thus, the role of QS in invasive staphylococcal infections can be questionable (Painter et al. 2014).

The data on c-di-GMP involvement in *S. aureus* biofilm formation seems to be controversial. Ishihara and coworkers (2009) suggested that this nucleotide is important for biofilm formation because a mutation in GdpS protein containing the GGDEF domain characteristic for diguanylate cyclases hampers its development. This defect was complemented by the addition of external c-di-GMP in physiological concentration. Opposite results were obtained by Holland and coworkers (2008) who demonstrated that, however, GdpS does, in fact, affect biofilm formation, but the mechanism of its activity is independent of c-di-GMP. The reason for the reported discrepancy can be due to physiological differences between the strains used. It was also shown that the treatment of *S. aureus* with extracellular c-di-GMP applied in high concentration, ranging between 0.02 and 0.2 μM, suppressed biofilm formation by the inhibition of intercellular adhesive interactions (Karaolis et al. 2005). These authors even suggested that c-di-GMP can be used as a novel anti-biofilm agent.

In *S. aureus*, 250 sRNA genes were discovered; however, functional studies are still lagging behind (Romilly et al. 2012). It was shown that the 3’ domain of the already mentioned RNAIII transcript represses, at the post-transcriptional level, the synthesis of cell-wall hydrolytic enzymes and, thus, negatively influences biofilm formation (Boisset et al. 2007). In a similar way, the 3’ untranslated domain of *icaR* transcript encoding a transcriptional repressor of biofilm polysaccharide synthesis interferes with the translation initiation of its own RNA (Ruiz de los Mozos et al. 2013). It was also shown that the 5’ untranslated, 196 nucleotides long, region of *sarA* transcript, designated teg49, induces the formation of biofilm through the positive regulation of the *sar* locus encoding SarA protein, promoting the initial steps of biofilm formation (Kim et al. 2014).

Two other major factors relevant for biofilm development in *S. aureus* are SarA (staphylococcal accessory regulator) and SigB. The *sarA* transcript is upregulated in biofilm when compared to planktonic cultures (Beenken et al. 2003) and its product inhibits the expression of nuclease Nuc and also proteases, thus preventing the degradation of biofilm structural components (Tsang et al. 2008). It was shown that SarA regulates the expression of cell wall-associated and certain extracellular proteins in *agr*-dependent and *agr*-independent pathways (Arya and Princy 2013). In turn, an alternative sigma factor, SigB (Kullik and Giachino 1997), is involved in the early stages of biofilm formation. It was shown that *sigB*-deficient *S. aureus* does not form a biofilm and upregulates RNAIII, which promotes the antibiofilm Agr system (Rachid et al. 2000).

**Fig. 4:**
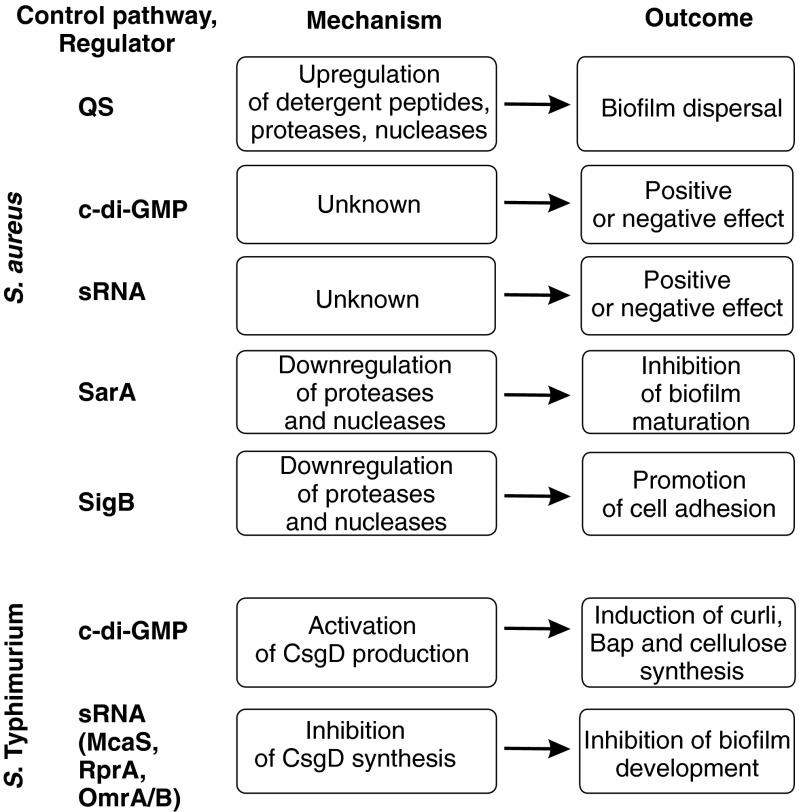
Control of biofilm formation in *S. aureus* and *S.* Typhimurium. *S. aureus*: biofilm control by five main regulatory factors is shown. QS positively regulates the synthesis of detergent-like peptides, proteases, and nucleases, resulting in biofilm dispersal. The involvement of c-di-GMP and sRNAs in biofilm regulation is still controversial, and the mechanism of their activity is unknown. SarA inhibits the expression of proteases and nucleases and, thus, promotes the development of immature biofilm. Alternative sigma factor SigB promotes the expression of adherence factors and, thus, positively regulates the initial steps of biofilm formation. *S.* Typhimurium: c-di-GMP activates the master CsgD and, subsequently, increases the synthesis of curli, Bap, and cellulose. Several sRNAs (McaS, RprA, OmrA/B, and possibly GcvB) inhibit the translation of CsgD mRNA and inhibit biofilm development

The factors involved in *S. aureus* biofilm formation, maintenance, and detachment are presented in Fig. 4.

## Biofilms of the enteric bacteria *Salmonella* Typhimurium and *Vibrio cholerae*

Gastrointestinal diseases usually arise upon the ingestion of food or water contaminated by enteric bacterial pathogens (Fàbrega and Vila 2013). Microorganisms frequently associated with illness are *Salmonella enterica* serovars, *Escherichia coli* pathovars, *Shigella* spp., *Campylobacter* spp., *Yersinia enterocolitica*, *V. cholerae*, and *Listeria monocytogenes*. The genetics of biofilm formation and dispersal are the most intensively studied in *S. enterica* sv. Typhimurium and *V. cholerae*.

### *Salmonella enterica* serovar Typhimurium

The ability to form a biofilm is an important factor of *Salmonella* virulence. For example, *S. enterica* serovar Typhi frequently forms biofilms on gallstones, resulting in a chronic infection of the gall bladder and the development of *Salmonella* carrier state—a serious public health problem (Gonzalez-Escobedo et al. 2011). Striking similarities in biofilm composition and regulatory circuits between several serovars of *S. enterica* and *E. coli* have been shown. The control of various stages of biofilm development is the best studied in *S. enterica* sv. Typhimurium, the most common causal agents of gastrointestinal diseases.

*S.* Typhimurium biofilm matrix is composed of proteins and exopolysaccharides. A major protein component is curli (amyloid fimbriae), encoded by *csg* operons (Yaron and Römling 2014). Protein BapA constitutes another important component of the matrix (Barnhart and Chapman 2006) and major biofilm exopolysaccharides are cellulose (Zogaj et al. 2001) and colonic acid (Gibson et al. 2006). Adhesion-mediated type I fimbriae, Lpf and Pef, also contribute to the early steps of biofilm formation (Ledeboer et al. 2006).

Biofilm formation is controlled by the master regulator, CsgD protein, belonging to the LuxR family of regulators. *csgD* expression is positively regulated by an alternative sigma factor, σ^s^; thus, the level of CsgD is high in the stationary phase of growth (Yaron and Römling 2014). CsgD increases curli and Bap expression and also, post-transcriptionally, indirectly activates cellulose biosynthesis (Fàbrega and Vila 2013). In the regulation of *S.* Typhimurium biofilm, c-di-GMP and sRNA play a crucial role. The elevated level of CsgD activates the transcription of the *adrA* gene encoding diguanylate cyclase synthesizing signal molecule c-di-GMP. In turn, c-di-GMP activates *csgD* expression in a complex way, involving at least eight GG(D/E)EF/EAL domain proteins (Ahmad et al. 2011; Anwar et al. 2014; Römling 2012). Moreover, c-di-GMP modulates cellulose biosynthesis (Latasa et al. 2005) and is responsible for the so-called rdar morphotype (red, dry, rough), characteristic for a potent biofilm producer (Ahmad et al. 2011). In addition to c-di-GMP, CsgD synthesis is also regulated at the post-transcriptional level by sRNAs. *csgD* mRNA is a direct target for several sRNAs, McaS, RprA, OmrA/OmrB, and possibly GcvB. All these sRNAs negatively regulate CsgD synthesis by binding to the overlapping 5’-region of the transcript, masking the ribosome binding site and, thus, inhibiting translation or inducing mRNA degradation (Mika and Hengge 2013). The principles of the regulation of *S.* Typhimurium biofilm are outlined in Fig. 4.

### *Vibrio cholerae*

*V. cholerae* is a ubiquitous bacterium in aquatic systems but also causes cholera, a severe diarrheal disease resulting from the consumption of contaminated drinking water (Faruque et al. 1998). It has a capacity to form biofilm, in both aquatic ecosystems and within the host (Watnick and Kolter 1999). The initial stages of biofilm formation are promoted by flagella-mediated motility and three types of pili (Yildiz and Visick 2009). The biofilm matrix is composed of *Vibrio* polysaccharide (VPS) containing glucose and galactose and minor constituents, N-acetyl glucosamine, mannose, and xylose (Yildiz and Schoolnik 1999), and matrix proteins RbmA, RbmC (rugosity and biofilm structure modulators), and Bap1 (Berk et al. 2012). The positive regulators of VPS production are the proteins VpsR and VpsT, which promote the transcription of *vps* structural genes (Yildiz et al. 2004; Casper-Lindley and Yildiz 2004). Biofilm formation in *V. cholerae* is regulated by QS, c-di-GMP, and sRNA. Very tight regulatory connections between these three factors have been proven (Srivastava and Waters 2012).

**Fig. 5:**
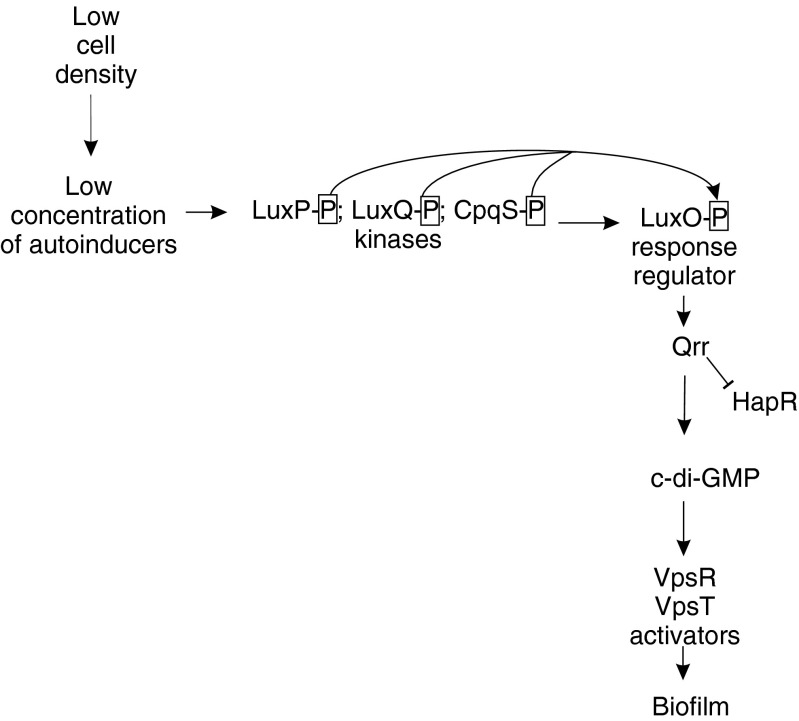
Control of biofilm development in *Vibrio cholerae* at low cell density. At low concentration of autoinducers, histidine kinases LuxP and CpqS are phosphorylated and able to phosphorylate regulator LuxO. LuxO–P activate the expression of Qrr 1–4 RNAs, which, in turn, positively influence the level of c-di-GMP. c-di-GMP activates VpsR and VpsT proteins, which positively regulate biofilm genes

The formation of *V. cholerae* biofilm is induced at low cell density and repressed at high cell density (Ng and Bassler 2009). The *Vibrio* QS system is composed of two sensory circuits that respond to two different autoinducers: Als–AI-2 or a hydroxylated alkyl ketone, CAI-1 (Higgins et al. 2007; Tiaden et al. 2010). At low concentrations of inducers, the periplasmic receptors, respectively, histidine kinases LuxPQ and CpqS, phosphorylate the response regulator LuxO. When phosphorylated, LuxO activates the expression of four small RNAs, Qrr 1–4 (quorum-regulated RNAs). These RNAs are transcriptionally activated not only by LuxO–P but also by an alternative σ^54^, and their activity appears to require the RNA-binding chaperone Hfq (Bardill and Hammer 2012; Lilley and Bassler 2000). Qrrs repress the synthesis of protein HapR, a negative regulator of c-di-GMP synthesis, and, at the same time, enhance the production of c-di-GMP synthesizing enzymes. Control of the c-di-GMP level by QS and Qrr constitutes a complicated circuit, moreover in that *V. cholerae* contains 61 predicted enzymes involved in the synthesis/degradation of this nucleotide (Galperin 2004). Summarizing, the activity of Qrr results in high c-di-GMP level at low cell density. c-di-GMP positively controls biofilm development by binding to VpsR and VpsT, which are the direct activators of biofilm genes (Hammer and Bassler 2003; Vance et al. 2003; Srivastava and Waters 2012). The control of biofilm formation in *V. cholerae* is schematically presented in Fig. 5.

At high cell density, the interaction of inducers and receptors switches their activity to phosphatases and LuxO is dephosphorylated. As a result, Qrr level decreases, HapR protein is synthesized, c-di-GMP level drops, and biofilm formation is inhibited (Tu and Bassler 2007). Repression of biofilm formation at high cell density could lead to the dispersal of mature *V. cholerae* biofilm (Srivastava and Waters 2012). Histone-like protein, H-NS, is another factor involved in the negative control of *V. cholerae* biofilm (Teschler et al. 2015).

It was also shown that, in addition to c-di-GMP, other nucleotides control *V. cholerae* biofilm; cAMP represses biofilm formation (Liang et al. 2007), while ppGpp positively regulates this process (He et al. 2012).

## Targeting genetic determinants as a mode of biofilm modulation

A study aimed to discover new promising agents and strategies against bacterial infections, especially those associated with biofilms, is an urgent task due to biofilm resistance to already used antimicrobial agents. Innovative anti-biofilm strategies are the subject of recent reviews (Cushnie and Lamb 2011; Joo and Otto 2012; Römling and Balsalobre 2012; Markowska et al. 2013; Chung and Toh 2014; Masák et al. 2014; Tan et al. 2014).

QS inhibition, also called quorum quenching, QQ, is considered one of the promising anti-biofilm strategies. However, the recent view of the role of QS in infections, pointing to its importance in biofilm dispersal, provides an argument against the practical use of the QQ approach to cure diseases caused by biofilm (Otto 2014). Many compounds, both natural and synthetic, affect bacterial QS systems (Kalia 2013), thus influencing biofilm development. Two of them, vanillin and cinnamic acid, were even shown to stimulate the formation of biofilm due to their ability to induce AHL synthesis (Plyuta et al. 2013). Unfortunately, up to 2014, only two clinical trials on QS inhibitors have been performed (Scutera et al. 2014).

The ideal QS inhibitors should fulfill the following criteria: (i) they should be low molecular weight and stable compounds, (ii) their activity should be highly specific and not toxic for the eukaryotic hosts, (iii) they should not interfere with the basal metabolic processes that can be targets for the development of drug resistance (Bhardwaj et al. 2013).

There are several potential classes of QS inhibiting strategies. One class targets QS signal production. For example, it was demonstrated that the analog of methylthioadenosine/S-adenosylhomocysteine nucleosidase (MTAM) blocks AI-1 and AI-2 acyl-lactone-based signal molecules (Gutierrez et al. 2009), methyl anthranilate inhibits the production of PQS (Calfee et al. 2001), and eugenol, the major compound of clove extract, decreases the transcriptional activation of *P. aeruginosa las* and *pqs* systems (Zhou et al. 2013). The second group of strategies neutralizes QS signals by enzymatic or antibody-mediated inactivation (Kalia and Purohit 2011). QQ enzymes can either hydrolyze the AHL molecules, e.g., AHL-lactonase, or reduce carbonyl to hydroxyl groups by the activity of oxidoreductases (Scutera et al. 2014). Long-acyl AHLs are degraded with the participation of AHL-acylase (Huang et al. 2003). The immunological approach includes the use of monoclonal antibodies, such as AP4-24H11, against *S. aureus* autoinducer (Park et al. 2007) or antibodies against *P. aeruginosa* homoserine lactones (Palliyil et al. 2014). Another class of strategies explores compounds whose targets are autoinducer–receptor interactions and/or receptor-mediated signals. Synthetic brominated derivatives of furanone known as C-30, AHL analogs, hamamelitannin, and also the extracts of several common fruits, herbs, and spices inhibit QS receptors, LasR and RhlR (Vattem et al. 2007; Sintim et al. 2010; O’Loughlin et al. 2013). For example, clove oil inhibits Las- and Rhl-regulated virulence factors, swimming motility, and extracellular polymeric substance (EPS) production in *P. aeruginosa* (Husain et al. 2013). In turn, solonamides isolated from *Photobacterium*, due to their structural similarity to *agr* AI, competitively inhibit the *agr* system in *S. aureus* (Mansson et al. 2011). Yet another compound, a sesquiterpene alcohol, farnesol, produced by *Candida albicans* and also present in the essential oils of citrus fruits, affects biofilm formation by *P. aeruginosa*, *C. albicans*, *S. aureus*, and *Streptococcus mutans* by interfering with the transcription of QS operons (Cugini et al. 2007; Jabra-Rizk et al. 2006). A number of compounds were identified that target the response regulator LuxO and the transcriptional repressor HtpR in *V. cholerae*. One of the most potent is pyrrole analog precursor of cholerae autoinducer 1, CA1 (Perez et al. 2014).

Several QS inhibitors can also enhance the activity of existing antibiotics and restore immune response efficiency (Jakobsen et al. 2012). For example, it was demonstrated that the QS inhibitor, hamamelitannin, acts synergistically with vancomycin or clindamycin against *S. aureus* (Brackman et al. 2011). It was also shown that biofilms treated with synthetic furanone C-30 are susceptible to tobramycin and readily dispersed by detergents (Hentzer et al. 2003). Moreover, QS mutants and cells treated with QS inhibitors were found to be prone to oxidative burst and phagocytosis (Bjarnsholt et al. 2005).

In our group, the trial was performed to evaluate the inhibitory activity of nanosilver on the QS system in *P. aeruginosa*. Silver nanoparticles were chosen due to their strong antibacterial activity, pleiotropic effect on bacterial cell, and lack of documented resistance development (Markowska et al. 2013). Unfortunately, no inhibition of QS LasI and Rhl1 systems was determined (data not published).

Some researchers stress the advantages connected with the use of QQ compounds. Because QS inhibitors do not exert a direct bactericidal effect, it is commonly considered that there is less selection pressure and less likelihood of resistance development (Pan and Ren 2009). Unfortunately, recent evidence indicates the development of the resistance to QS inhibitors in *P. aeruginosa* (Kalia et al. 2014). The increased resistance exploits the efflux mechanisms, e.g., *mexR* and *nalC* mutants demonstrated the increased resistance to C-30 (Maeda et al. 2012). The potential risk connected with the use of all QQ strategies described above should also be mentioned. QS inhibitors can select more virulent strains by interfering with natural selection towards reduced virulence (Köhler et al. 2010). Moreover, the elimination of a particular pathogen can predispose patients to be infected with others.

As the prominent role of c-di-GMP is the activation of biofilm formation, the signals that downregulate its concentration in a cell can be considered potential anti-biofilm agents. There are several approaches to interfere with c-di-GMP signaling, e.g., manipulation with enzymatic activities, interference with signal perception, and direct inactivation (Römling and Balsalobre 2012). The inhibition of c-di-GMP synthesizing activity or stimulation of phosphodiesterase activity can diminish or enhance biofilm formation, respectively (Chávez de Paz et al. 2012; An et al. 2010). There are no natural compounds interfering with the synthesis or activity of c-di-GMP, but among synthetic compounds, sulfathiazole and N-(4-aminophenyl) benzamide were shown to be potent inhibitors of biofilm formation, this being achieved by suppressing diguanlyate cyclases (Antoniani et al. 2010). Several inhibitors targeting diguanylate cyclases, named DCG inhibitors, and, thus, influencing c-di-GMP metabolism in *V. cholerae* were identified (Sambanthamoorthy et al. 2012, 2014). In turn, azathioprine interferes with intracellular nucleotide pool availability (Antoniani et al. 2013). Other signals controlling the c-di-GMP level can also be used to regulate c-di-GMP signaling. For example, *P. aeruginosa* exposure to nitric oxide (NO) stimulates c-di-GMP-specific phosphodiesterase activity, thus promoting biofilm dispersal (Barraud et al. 2009), whereas the sequestration of c-di-GMP by high-affinity receptors removes the available nucleotide pool and promotes biofilm dispersal, phenotypically mimicking phosphodiesterase activity (Ma et al. 2011). c-di-GMP stimulates biofilm development only in a relatively narrow range of concentration; it was shown that extracellular c-di-GMP, when applied in high concentration, acts as an inhibitor of biofilm formation by *S. aureus* (Karaolis et al. 2005). It should also be mentioned that c-di-GMP and other cyclic di-nucleotides can serve as potential adjuvants, and their high efficacy to stimulate an immune response can constitute a future strategy to inhibit biofilm formation (Karaolis et al. 2007).

Targeting the regulatory sRNAs is another potential way to modulate the expression of genes important for biofilm development (Kang et al. 2014). Experiments performed with *E. coli* have shown that the modulation of expression of several sRNAs, OmrR, OmrB, and McaS, leading to the change in cell motility, production of curli, and export of exopolysaccharides, results in the inhibition of biofilm formation. Also, the knockout of other sRNAs, Arc2, SdsR, GadY, and MicA, affects biofilm development and motility, although their mode of action remains elusive (Mandin and Guillier 2013). Metabolic engineering and the possibility to synthesize artificial RNAs of choice (Man et al. 2011) create the opportunity for the silencing of any specific gene and, therefore, inhibit various steps of biofilm formation or enhance biofilm dispersal.

## Concluding remarks

The biofilm formation and dispersal in pathogenic bacteria has been studied extensively, and a large number of literature positions dealing with these processes, including their genetic regulation, has been published. The regulators being the subject of this paper, QS, c-di-GMP, and sRNA, are necessary for biofilm biology in all four described bacterial species, but the details of their regulatory role and the importance of the particular factor vary between species.

In *P. aeruginosa* and *S. aureus*, QS systems regulate mainly biofilm dispersal, while in *V. cholerae*, QS is important for biofilm formation, and it was proved that this process is induced at low cell density. There are no available data on the regulation of *S.* Typhimurium biofilm by QS. In turn, c-di-GMP signaling is a factor regulating biofilm formation in *P. aeruginosa*, *S.* Typhimurium, and *V. cholerae*, but its involvement in the regulation of *S. aureus* biofilm is still controversial. The interactions between QS and c-di-GMP regulatory pathways exist and are especially well documented for *V. cholerae*. Finally, as the variety of sRNAs regulates the large spectrum of bacterial genes, it can be expected that these molecules can also be involved in biofilm biology. Indeed, sRNAs have been shown to regulate post-transcriptionally, usually negatively, biofilm formation in all four described species. The interconnections between sRNAs and other regulators are proved.

The number of attempts to target genetic determinants in order to modulate biofilms formed by bacterial pathogens is growing exponentially. In the in vitro experiments, several small-molecule therapeutics were discovered. They can be divided into four classes: QS inhibitors, disruptors of c-di-GMP signaling, inhibitors of sRNAs activity, and compounds with unknown target. The technique combining microscopic imaging with cellular viability measurements allows to identified the compound that selectively disrupt biofilm formation without affecting cell survival (Teschler et al. 2015). All compounds giving positive results during the in vitro studies should pass preclinical and clinical trials in order to be accepted in the therapy arena.
